# Clinical Profile, Antibiotic Resistance and Outcomes in Bacterial Endophthalmitis: Coagulase-Negative Staphylococcus Endophthalmitis as Compared to Other Organisms

**DOI:** 10.7759/cureus.53532

**Published:** 2024-02-04

**Authors:** Nripen Gaur, Brijesh Takkar, Parijat Chandra, Somya Puri, Gita Satpathy, Yog R Sharma

**Affiliations:** 1 Ophthalmology, All India Institute of Medical Sciences, Bilaspur, Bilaspur, IND; 2 Smt Kanuri Santhamma Centre for Vitreoretinal Diseases, L V Prasad Eye Institute, Hyderabad, IND; 3 Vitreo-retinal Services, Ophthalmology, All India Institute of Medical Sciences, New Delhi, New Delhi, IND; 4 Microbiology, All India Institute of Medical Sciences, New Delhi, New Delhi, IND

**Keywords:** coagulase-negative staphylococcus endophthalmitis, gram-negative endophthalmitis, antibiotic resistance, endophthalmitis, staphylococcus

## Abstract

Background: To evaluate the clinical profile, outcomes and antibiotic resistance in bacterial endophthalmitis.

Methods: This was a post-hoc analysis of a study conducted at a tertiary centre, where 60 consecutive cases of culture-proven bacterial endophthalmitis were included prospectively. Group 1 included coagulase-negative Staphylococcus endophthalmitis (CNSE), while group 2 included the remaining cases. Clinical features, antibiotic resistance and visual outcomes were compared. Visual acuity >3/60 at six months of follow-up was defined as a good visual outcome.

Results: Group 1 had 31 cases, while group 2 had 29. Group 2 included 12 gram-positive and 17 gram-negative isolates. Among the groups, group 2 had more patients with presenting visual acuity below hand motions close to the face (25 vs. 12, p<0.001), poor visual outcomes (26 vs. 3, p<0.001) and retinal detachment (RD) (10 vs. 2, p=0.007). Pseudomonas was most commonly resistant to antibiotics, and ceftazidime (p=0.005) and cefazolin (p=0.009) resistance were higher in group 2 isolates. In group 1, five isolates were resistant to any one of the antibiotics, whereas in group 2, 13 isolates were resistant to any one of the antibiotics (p=0.024).

Conclusions: In the current study, eyes in the group of endophthalmitis caused by CNSE achieved better visual acuities at the last follow-up compared to eyes with endophthalmitis caused by other bacteria. Antibiotic resistance in isolates other than CNSE is a cause of concern.

## Introduction

Infective endophthalmitis is a serious complication, detrimental to the visual and structural outcomes of the eye. Post-cataract surgery endophthalmitis (PCE) is the most common type, with *Staphylococcus epidermidis* being the most common microbe implicated in PCE. The results of the Endophthalmitis Vitrectomy Study showed that 68% of culture-positive isolates were coagulase-negative Staphylococcus [1]. However, *Staphylococcus epidermidis* is also known to be the predominant microbe in traumatic endophthalmitis and is listed among the organisms causing endogenous endophthalmitis [2,3].

A very important factor contributing to the outcome of any infective process localised to a compartment is the virulence of the microbe [4]. Yet, the outcomes and clinical profiles of PCE and post-traumatic endophthalmitis (PTE) can be very contrasting. However, this may be linked to other factors that accompany trauma like foreign body (FB) [5] and preoperative prophylactic measures, as pointed out by the ESCRS study [6]. Not only do such measures decrease infection rates, but they also suppress ocular colonisation by the inoculated microbes, thus dampening the damage. The ocular surface as well as the adnexa are important sources of infection in post-operative endophthalmitis. Blepharitis, conjunctivitis, canaliculitis, lacrimal duct obstructions, and contact lens wear are amongst the peri-operative risk factors for post-operative endophthalmitis. The use of contaminated agents or surgical equipment perioperatively may also cause infection.

This is a post-hoc analysis of our previously published study on predictors of visual outcomes in endophthalmitis, which showed a high incidence of *Staphylococcus epidermidis* at our place, as much as 72.93% amongst gram-positive bacteria [7]. In this study, we analyse the severity of coagulase-negative Staphylococcus endophthalmitis (CNSE) in terms of clinical profile and visual outcomes as compared to other bacteria.

## Materials and methods

This is a secondary analysis of a study conducted at a tertiary eye care centre in Northern India. The details of the original study have been published elsewhere. Institute review board ethical clearance was obtained. The study was conducted in accordance with the Declaration of Helsinki, and informed consent was obtained for all procedures and investigations.

Consecutive patients presenting with a clinical diagnosis of infective endophthalmitis to the emergency and vitreo-retina services of our centre from April 2013 to March 2014 were analysed prospectively. Patients with endogenous endophthalmitis, culture-negative results and culture-proven fungal endophthalmitis were removed from the analysis. Detailed ophthalmic workup was done for all the patients and clinical profile, inclusive of presenting visual acuity, symptoms, signs, complications and outcomes, was recorded. Visual acuity was carefully noted by a single investigator using the Endophthalmitis Study (EVS) guidelines [8]. Extensive media haze was defined as the inability to visualise the optic disc on indirect ophthalmoscopy. The anterior segment signs were recorded using a slit lamp biomicroscope. Ultrasound was done for all the infected eyes, and imaging was done for FB as needed. Eyes with visual acuity better than hand motions close to the face and those where surgery was not possible, i.e., with corneal abscess, opacity or oedema precluding vitrectomy, were treated with intravitreal antibiotics (ceftazidime (2.25 mg/0.1 ml) and vancomycin (1 mg/0.1 ml)). Three-port vitrectomy was done for the rest along with the injection of intravitreal antibiotics at the end of the procedure on the day of presentation itself without any delay. In all the cases, a vitreous sample was obtained before the procedure with a vitreous tap/cutter. These were evaluated with gram stain and KOH mount and inoculated in blood agar, chocolate agar, thioglycolate broth and Sabouraud dextrose agar without cycloheximide. Anaerobic culture was not done routinely. After the procedure, all patients received fortified topical cefazolin (5%) and tobramycin (1.3%) once every two hours and oral ciprofloxacin (dose adjusted as per weight, 500 mg twice a day for 10 days for healthy adults) initially. A further treatment plan was decided depending on the clinical course. No patient had received intravitreal steroids. All the patients were followed up for a minimum period of six months.

Finally, 60 patients with culture-proven bacterial endophthalmitis were included for statistical analysis (SPSS Software, version 16, IBM Corp., Armonk, New York, USA). Inoculums growing coagulase-negative Staphylococcus were grouped as group 1, while the rest were grouped as group 2. Visual acuity at six months of follow-up was analysed into three groups: < hand motions close to face (HMCF), HMCF to 3/60, and >3/60. A good visual outcome was defined as a final best corrected visual acuity (BCVA) >3/60. The two groups were compared for clinical presentation, outcomes and drug resistance to antibiotics using the Chi-square test. A two-tailed P-value less than 0.05 was taken as significant.

## Results

The mean age of the patients included for analysis was 37.63 ± 23.83 years. On subgroup analysis, the mean age was 34.51 ± 23.34 years in group 1 and 40.96 ± 24.31 years in group 2. Overall, 36 patients (60%) were male. The clinical diagnosis included 27 cases of post-cataract endophthalmitis (PCE) and 29 cases of post-traumatic endophthalmitis (PTE). Four other patients included two cases of bleb-related endophthalmitis, one post-intravitreal injection and one post-keratitis endophthalmitis. Overall, the initial presenting vision was <HMCF in 37 patients (61.7%). At six months of follow-up, 32 (53.3%) had a final vision of >3/60. Four of the 29 PTE patients were found to have an ocular FB (two each in both groups).

About 43 (71.7%) isolates were gram-positive, while 17 (28.3%) were gram-negative. No case had mixed infection. About 31 (51.7%) isolates were found to be positive for coagulase-negative Staphylococcus. The second group had 12 gram-positive and 17 gram-negative bacteria. Among these, we isolated nine cases of Pseudomonas species, eight cases of *Staphylococcus aureus*, four cases of *Streptococcus pneumonia*, three cases each of Klebsiella species and Acinetobacter species, and one case each of *Escherichia coli* and Enterobacter species. On comparing groups 1 and 2, group 2 was found to have significantly more patients with BCVA<HMCF (p<0.001), retinal detachment (RD) (p=0.007) and poorer visual outcomes (p<0.001). Details of the comparison between groups 1 and 2 are presented in Table 1. A detailed description of the outcomes of the group 2 isolates has been presented in Table 2.

**Table 1:** Clinical features in bacterial endophthalmitis.

Clinical features between groups 1 and 2			
Clinical features	Group 1 (N=31), n (%)	Group 2 (N=29), n (%)	P-value
Vision at presentation			
≤HMCF	12 (38.7)	25 (86.2)	<0.001
≤2/60	19 (61.3)	3 (10.3)	
>3/60	0 (0.0)	1 (3.4)	
Type of endophthalmitis			
Post-cataract surgery	14 (45.2)	13 (44.8)	0.089
Post-traumatic	17 (54.8)	12 (41.4)	
Others	0 (0.0)	4 (13.8)	
Hypopyon	28 (90.32)	23 (79.31)	0.292
Corneal infiltrate	2 (6.5)	2 (6.9)	>0.999
Fundus visibility	2 (6.5)	1 (3.4)	>0.999
Retinal detachment	2 (6.5)	10 (34.5)	0.007
Final vision >3/60	28 (90.3)	3 (10.3)	<0.001
HMCF: Hand motions close to face			

**Table 2:** Comparison between group 2 organisms. HMCF: hand motions close to face.

	Gram-positive (n=13)	Gram-negative (n=16)	P-value
BCVA < HMCF on presentation	11	14	>0.999
BCVA on presentation >3/60	0	1	
Visual outcome	5	5	>0.999
Visual outcome >3/60	1	2	

Overall, 42 (70%) isolates were susceptible to all the antibiotics administered. More than 80% of isolates were susceptible individually to all the antibiotics used: 50 patients each to ciprofloxacin and ceftazidime, 54 patients to cefazolin, and 57 patients each to vancomycin and tobramycin. On comparing groups 1 and 2, more isolates were susceptible to all the antibiotics used in group 1, but statistically significantly only to the cephalosporins. Details of this comparison have been presented in Table 3. In group 1, 5/31 isolates were found to be resistant to any one of the antibiotics used, whereas in group 2, 13/29 isolates were found to be resistant to any one of the antibiotics (p=0.024). None of the *Pseudomonas* sp. isolates was susceptible to ceftazidime. Five organisms in both groups were resistant to ciprofloxacin.

**Table 3:** Antibiotic sensitivity in bacterial endophthalmitis.

Antibiotic sensitivity			
Antibiotic	Group 1, n (%)	Group 2, n (%)	P-value
Ceftazidime	30 (96.8)	20 (69)	0.005
Vancomycin	30 (96.8)	27 (93.1)	0.606
Ciprofloxacin	26 (83.9)	24 (82.8)	>0.999
Tobramycin	31 (100)	26 (89.7)	0.107
Cefazolin	31 (100)	23 (79.3)	0.009

In group 1 patients, on analysis of the impact of the mode of inoculation on clinical factors and visual outcomes, no statistically significant association could be identified (Table 4).

**Table 4:** Impact of the mode of inoculation in Staphylococcus epidermidis-related endophthalmitis.

Impact of the mode of inoculation on clinical factors and visual outcomes			
	PCE (N=14)	PTE (N=17)	P-value
Vision at presentation			
	6 (42.8%)	6 (35.3%)	0.724
>HMCF	8 (57.2%)	11 (64.7%)	
Hypopyon			
Yes	12 (85.7%)	16 (94.1%)	0.576
No	2 (14.3%)	1 (5.9%)	
Corneal infiltrate			
Yes	2 (14.3%)	0 (-)	0.196
No	12 (85.7%)	17 (100%)	
Fundus visibility			
Yes	0 (-)	2 (11.8%)	0.488
No	14 (100%)	15 (88.2%)	
Retinal detachment			
Yes	2 (14.3%)	0 (-)	0.196
No	12 (85.7%)	17 (100%)	
Final vision >3/60			
Yes	12 (85.7%)	17 (100%)	0.196
No	2 (14.3%)	0 (-)	
PCE: post-cataract surgery endophthalmitis, PTE: post-traumatic endophthalmitis, HMCF: hand motions close to face			

## Discussion

In this study, we aimed to evaluate the clinical profile, outcomes and antibiotic resistance in bacterial endophthalmitis. The findings of Tables 1, 3 indicate better visual outcomes and less antibiotic resistance in CNSE as compared to other isolates causing endophthalmitis.

In any infective condition, promptness and accuracy of antibiotic therapy play a very crucial role in salvaging organ function, more so in the microbial invasion of compartments like the eye. Thus, the choice of empirical antibiotics is important in endophthalmitis [9,10], while awaiting the laboratory results. It is well known that the spectrum of isolates is variable depending on the mode of inoculation. In PTE, commonly implicated isolates are *Staphylococcus epidermidis*, *Streptococcus pneumoniae*, *Staphylococcus saprophyticus* and *Bacillus* sp. [11-17]. In PCE, the spectrum is slightly different as *Staphylococcus epidermidis*, *Staphylococcus aureus* and *Pseudomonas* sp. are known to be common [7,10,12]. Mixed infections can also be seen in around 5% of cases [2,11]. Thus, the distribution of microbial isolates in the current study is as per the findings of the literature [11-17]. Knowledge of this spectrum is important because of changing patterns of microbial profile and drug sensitivity [9,16]. Because of its abundance in the conjunctival flora, CNSE is very common. The ability of the organism to effect colonisation while protecting itself from immune responses is believed to be because of the production of a biofilm (slime, an exo-polysaccharide) [18-21]. Many studies have been conducted to assess therapies for the management and prophylaxis of CNSE endophthalmitis [22-30].

CNSE had not only a better presentation but also better outcomes than group 2. In the parent study of this research, we also found cases with better presenting visual acuity to have better visual results [7]. Although gram-negative bacteria are associated with poor visual results in endophthalmitis [9], this may not always be so. The results from Table 2 suggest that after the exclusion of coagulase-negative Staphylococcus, results were similar for gram-positive and gram-negative bacteria. Also, we could not assess any significant impact of the mode of inoculation on visual results amongst the group 1 patients (Table 4). We could not find any difference in clinical presentation either (Table 4). Results of Tables 2, 4 suggest that CNSE indeed has better visual results due to the inherent low virulence of the microbe. A previous retrospective study on 86 CNSE, due to all causes, also found the median visual acuity to improve to 20/60 [31]. However, the study did not compare the results with those of other bacteria. Future prospective studies should compare the mode of inoculation and virulence of microbes for their impact on visual outcomes.

In the current retrospective study, a greater number of retinal detachments were noted in group 2 cases (Table 1). Patients with RD had seven times higher odds of infection with bacteria other than coagulase-negative Staphylococcus (95% CI: 1.5 to 38.7). Although trauma is a risk factor for RD, this result becomes more prudent if one considers that coagulase-negative Staphylococcus patients predominated in the PTE group too (Table 1). One reason for this may be the severity of the disease caused by group 2 isolates, which may have led to either the formation of necrotic rhegma or sub-retinal exudation.

In a recent review of gram-positive organisms with reduced vancomycin sensitivity, only 27 cases were identified over 25 years [32]. Among these four were coagulase-negative Staphylococcus. A study from India on delayed-onset PCE found only 70% of the gram-negative isolates to be susceptible to ceftazidime [33]. A study over 14 years on PTE patients found 77% of gram-negative isolates to be susceptible to ceftazidime, with a very high susceptibility of gram-positive organisms to vancomycin [16]. Another 25-year review on microbial sensitivity to antibiotics found increasing resistance to cephalosporins. The authors had identified nearly 10% of gram-negative isolates to be resistant to ceftazidime [34]. They concluded that the combination of ceftazidime and vancomycin to be an excellent choice for empirical intravitreal therapy. In the current study, we identified a total of 3/60 isolates to be resistant to both intravitreal antibiotics. We also found group 2 isolates to be significantly resistant to both the cephalosporins used in the study upon comparison with coagulase-negative Staphylococcus (Table 3). Overall, only one gram-positive isolate was resistant to vancomycin, while all nine gram-negative isolates resistant to ceftazidime belonged to the genus Pseudomonas. Due to the changing patterns of microbial susceptibility to antibiotics [15], such studies need to be done regularly. 

Limitations

A major limitation of this study is its retrospective nature with the inherent information bias. Because of the heterogeneity of isolates in group 2, no direct comparisons were possible between different microbes. No differentiation was done in the current study for exudative and rhegmatogenous RD. As there was no case of mixed microbial infection, its impact on results could not be studied. Some results, as in Table 4, may have been masked by a smaller sample size.

## Conclusions

Endophthalmitis caused by coagulase-negative Staphylococcus has the most favourable visual outcomes amongst bacteria due to the low virulence of the organism. The presence of retinal detachment may indicate infection by microbes other than coagulase-negative Staphylococcus. Resistance to antibiotics is prevalent in other organisms, especially Pseudomonas, and needs regular monitoring. As a result of the rising antibiotic resistance, antibiotic stewardship to prevent the same is paramount.
